# Toll-Like Receptors and Myocardial Ischemia/Reperfusion, Inflammation, and Injury

**DOI:** 10.2174/157340309788970405

**Published:** 2009-08

**Authors:** David J Kaczorowski, Atsunori Nakao, Kenneth R McCurry, Timothy R Billiar

**Affiliations:** 1 Department of Surgery, University of Pittsburgh, Pittsburgh, PA, USA; 2 Thomas E. Starzl Transplant Institute, University of Pittsburgh, Pittsburgh, PA, USA; 3 Heart, Lung, Esophageal Institute, University of Pittsburgh Medical Center, Pittsburgh, PA, USA; 4 Heart and Vascular Institute, Cleveland Clinic, Cleveland, Ohio, USA

**Keywords:** Toll-like receptors, ischemia/reperfusion, transplant, inflammation, heart.

## Abstract

Cardiac ischemia/reperfusion (I/R) injury occurs in several important clinical contexts including percutaneous coronary interventions for acute myocardial ischemia, cardiac surgery in the setting of cardiopulmonary bypass, and cardiac transplantation. While the pathogenesis of I/R injury in these settings is multifactorial, it is clear that activation of the innate immune system and the resultant inflammatory response are important components of I/R injury. Toll-like receptor 4 (TLR4), originally identified as the sensor for bacterial lipopolysaccharide (LPS), has also been shown to serve as a sensor for endogenous molecules released from damaged or ischemic tissues. Accordingly, recent findings have demonstrated that TLR4 not only plays a central role as a mediator of cardiac dysfunction in sepsis, but also serves as a key mediator of myocardial injury and inflammation in the setting of I/R. Furthermore, TLR4 may play a role in the development of atherosclerotic lesions. Other studies have implicated TLR4 in the adverse remodeling that may occur after ischemic myocardial injury. This emerging body of literature, which is reviewed here, has provided new insight into the early molecular events that mediate myocardial injury and dysfunction in the setting of I/R injury.

## TOLL-LIKE RECEPTORS (TLRS)

TLRs are a family of evolutionarily conserved receptors that play a critical role in activation of the innate immune system in response to microbial invaders. In 1996, Lamaitre and colleagues first reported that a *Drosophila* protein known as Toll, which was known to play a role in the development of the fly, was also an important mediator of the host immune response against fungal infection in the adult fly [1]. Medzhitov and Janeway later demonstrated that a homologous receptor (later identified as TLR4) exists in higher organisms including humans [2]. As many as eleven human and thirteen murine TLRs have subsequently been identified to date.

These receptors each contain a ligand binding domain with multiple leucine rich repeats, a transmembrane domain, and a Toll/IL-1R (TIR) signaling domain [3-5]. While they are similar in structure, TLRs differ both in their subcellular localization as well as the ligands that they recognize. For example, TLR1, -2, -4, -5, and -6 are predominantly located at the cell surface while TLR3, -7, and -9 are generally localized to the endosomal compartment in most inflammatory cell types. TLRs serve as pattern recognition receptors by sensing the presence of conserved microbial motifs and alerting the host to the presence of microbial invaders. TLR1, -2, and -6 detect microbial lipopeptides. TLR4, in conjunction with CD14 and MD2, senses the presence of lipopolysaccharide (LPS). Flagellin is the ligand of TLR5. TLR3 and -7 detect double stranded RNA and single stranded RNA, respectively, while TLR9 alerts the host to the presence of bacterial CpG DNA [6] and perhaps endogenous DNA motifs [7].

**Fig. (1):**
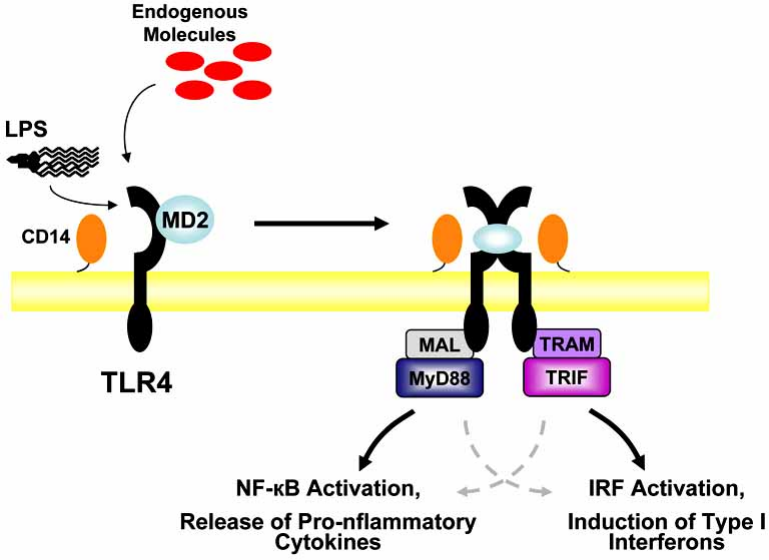
**TLR4 Signaling.** In conjunction with CD14 and MD2, TLR4 senses the presence of bacterial LPS and alerts the immune system to the presence of bacterial invaders. It has also been demonstrated that TLR4 can sense the presence of endogenous molecules that are released from damaged or ischemic tissues and initiate an inflammatory response. The roles of CD14 and MD2 in the detection of endogenous molecules are less clear. The molecular details of TLR4 signaling have not yet been entirely defined, but upon activation, it is likely that TLR4 clustering occurs. Intracellular adaptor proteins are then recruited and the signal is transmitted inside the cell, ultimately resulting in activation of the cell and a robust inflammatory response. TLR4 signaling through MyD88 ultimately results in NF-kB activation and release of pro-inflammatory cytokines while TLR4 signaling through the adaptor TRIF results in IRF activation and Induction of type I interferons. Some overlap may occur between these two pathways, as indicated here by dashed, gray arrows.

Upon TLR stimulation by corresponding ligands, intracellular adaptor proteins are recruited to the signaling complex and mediate downstream signaling [8,9]. Most TLRs, with the exception of TLR3, utilize the intracellular adaptor myeloid differentiation factor 88 (MyD88). After MyD88 is recruited to the signaling complex, downstream pathways are activated, culminating in the activation of nuclear factor-κB (NF-kB) [10]. TLR3 stimulation leads to recruitment of an adaptor known as TIR domain-containing-adaptor inducing IFNβ (TRIF) [11,12]. Through a number of downstream mediators, TRIF goes on to activate IRF3 and IRF7 and result in the production of Type I interferons. Interestingly, TLR4 can utilize both MyD88 and TRIF as adaptors for signaling (Fig. **1**). While these pathways are conceptually thought of as distinct, it should be noted that there is overlap between the two. TRIF-dependent signaling can result in activation of NF-kB [13,14] and conversely MyD88-dependent signaling can result in IRF activation [15].

TLR4 is one of the most thoroughly studied members of the TLR family. TLR4 was initially identified as the molecular sensor for bacterial LPS when Poltorak, Beutler, and colleagues demonstrated that the failure of C3H/HeJ and C57Bl/10ScCr mice to respond to LPS is due to mutations in the *tlr4* gene [16]. A number of other studies have also found that TLR4 can sense the presence of a number of endogenous molecules which can be released from damaged or ischemic tissues. Heparan sulfate, hyaluronan, fibrinogen, high mobility group box-1 (HMGB1) and others have been implicated as endogenous activators of TLR4 signaling [17-24]. Accordingly, TLR4 has been identified as a mediator of inflammation and organ injury in several models of sterile tissue injury including hemorrhagic shock [25,26] and femur fracture [27] as well as hepatic [28-31], renal [32], and pulmonary [33] warm I/R injury. 

## TLRS AND MYOCARDIAL DYSFUNCTION IN SEPSIS

Sepsis can result in pronounced myocardial dysfunction, characterized by contractile impairment of cardiomyocytes. Further, cardiomyocytes can adopt a pro-inflammatory phenotype, marked by production of pro-inflammatory cytokines and expression of adhesion molecules. Recent evidence implicates TLRs as mediators of myocardial dysfunction in the setting of sepsis.

Boyd and colleagues demonstrated that multiple TLRs, including TRL2, -3, -4, -5, -7, and -9 are expressed in murine heart tissue and a murine cardiomyocyte cell line [34]. The authors found that stimulation with ligands for TLR2, -4, or -5 results in impaired contraction of primary murine ventricular myocytes through a NF-κB-dependent mechanism. In addition, interleukin-6 (IL-6), keratinocyte-derived cytokine (KC), macrophage inflammatory protein-2 (MIP2) production and increased intercellular adhesion molecule-1 (ICAM-1) expression was observed after stimulation of a cardiomyocyte cell line with TLR ligands, particularly LPS. This also occurred in a NF-κB-dependent manner. The results of this study suggest that the cardiomyocyte itself is able to sense the presence of PAMPs through TLRs, and adopt a pro-inflammatory phenotype in response. Furthermore, this data argues that the contractile function of the cardiomyocyte can be directly modulated through TLR stimulation.

In a separate study, Baumgarten and co-investigators found that stimulation of ventricular myocytes with LPS resulted in reduced sarcomere shortening amplitude and prolonged duration of relaxation [35]. In contrast, contractile function was not impaired in cardiomyocytes from TLR4-mutant mice after stimulation with LPS. A soluble inhibitor of TLR4 was also able to prevent contractile dysfunction in wild-type cells. In accordance with the findings of Boyd and colleagues, these results suggest that stimulation of TLR4 on cardiomyocytes leads directly to contractile dysfunction. 

Tavener and colleagues also observed that mice experienced cardiac dysfunction after LPS injection [36]. However, they found that chimeric mice with TLR4-mutant leukocytes and TLR4 wild-type cardiomyocytes had no myocardial impairment in response to LPS, while mice with TLR4 wild-type leukocytes and TLR4 deficient cardiomyocytes demonstrated reduced myocyte shortening in response to LPS. These observations differ from those of Boyd [34] and Baumgarten [35], as they argue that TLR4 on immune cells, rather than cardiomyocytes, is responsible for myocardial impairment in response to LPS. Technical factors may be in part responsible for the different conclusions from these studies. Also, there may be a role for both myocardial and immune cell TLR4 signaling in cardiac dysfunction in response to LPS. However, additional study will be required to further clarify the mechanisms of TLR4-mediated myocardial dysfunction in sepsis

## TLR4 IN MYOCARDIAL WARM I/R

Warm myocardial I/R injury is encountered in settings including cardiac ischemia and subsequent restoration of blood flow with directed percutaneous interventions. Several investigators have explored the role of TLR4 in warm myocardial I/R. The results of multiple studies reveal that myocardial injury and associated inflammation is reduced in the absence of TLR4 signaling (Table **1**).

Using a temporary left anterior descending (LAD) artery occlusion model, Oyama and colleagues first observed reduced myocardial infarct size in 2 distinct strains of mice that lack functional TLR4 signaling [37]. Further, compared to those from wild-type animals, the authors observed reduced neutrophil infiltration as measured both by immunohistochemical staining and myeloperoxidase (MPO) activity in TLR4-deficient hearts. Accordingly, fewer lipid peroxidation products were measured in TLR4-deficient hearts after I/R. There was also less complement deposition in hearts from TLR4-deficient mice compared to controls after I/R, suggesting that TLR4 signaling might somehow influence local synthesis, activation, or deposition of complement.

These results were confirmed by Chong and co-investigators, who also utilized a similar regional warm myocardial I/R model to demonstrate that TLR4 is a mediator of myocardial injury and inflammation after I/R [38]. After I/R, Chong and colleagues found that TLR4 mutant mice exhibited significantly smaller infarcts. These investigators also found reduced activation of c-Jun N terminal kinase (JNK) and significantly lower transcript levels of a number of a number of pro-inflammatory cytokines, including IL-1β, IL-6, and monocyte chemotactic protein-1 (MCP-1). 

Furthermore, the phosphatidylinositol-3 kinase (PI3K) pathway may be partially responsible for the observed reduction in myocardial injury after warm I/R [39]. Pretreatment of animals with pharmacologic inhibitors of the PI3K/Akt signaling pathway, including either wortmannin or Ly294002, abrogated the observed reduction of myocardial infarct (MI) size in TLR4 deficient animals. Inhibition of the PI3/Akt pathway also increased apoptosis of cardiomyocytes after I/R. These effects appear to be independent of NF-κB, but increased phosphorylation of myocardial phosphatase and tensin homolog deleted on chromosome 10 (PTEN) and glycogen synthase kinase-3β may play a role. 

Collectively, these studies implicate TLR4 as a mediator of myocardial injury and inflammation after warm I/R. In each of these studies, animals with either a mutation in the signaling domain of the *tlr4* gene or lacking the gene encoding TLR4 altogether were used to study the role of TLR4 warm I/R of the heart. In order to evaluate the clinical potential of TLR4 inhibition in myocardial warm I/R, Shimamoto and co-investigators employed a compound known as Eritoran, a soluble analog of lipid A that serves as an inhibitor of TLR4 [40]. The authors found that pretreatment of animals with Eritoran significantly reduced infarct size in a murine model of regional myocardial I/R. Less phosphorylation of JNK and less NF-κB nuclear translocation was observed in hearts of animals that were pre-treated with Eritoran compared to those from control animals.

To better understand whether the intracellular adaptor MyD88 plays a role in TLR4-mediated myocardial I/R injury, Hua and colleagues transfected the myocardium of rats with an adenoviral construct containing a dominant negative MyD88 gene, which served to block MyD88 signaling. Inhibition of MyD88 signaling with the dominant negative MyD88 construct prevented NF-κB activation, stimulated PI3K/Akt pathway, and ultimately protected the myocardium from injury after I/R compared to controls [41]. These observations suggest that the MyD88-dependent pathway plays a role in warm I/R injury, but these findings do not exclude a role for the adaptor TRIF as a potential mediator of inflammation and organ injury after I/R.

## TLR4 IN MYOCARDIAL COLD I/R

In the process of solid organ transplantation, whole organs are flushed and stored in cold preservation solution and then subsequently re-implanted and reperfused with warm blood. I/R injury in solid organ transplantation is of particular interest, as it can affect graft function and may impact upon patient survival as well. 

Our group sought to determine whether TLR4 mediates the early inflammatory response and organ injury in a model of cold I/R. In order to address this question, we performed heterotopic cardiac transplants in both wild-type mice and mice deficient in TLR4 signaling due to a point mutation [42]. In wild-type mice, we observed significant elevations in the serum levels of a number of pro-inflammatory cytokines, particularly IL-6 and the chemotactic factor MCP-1. Tumor necrosis factor α (TNFα) and IL-1β levels were also elevated, but to a substantially lesser extent. Interestingly, lower systemic levels of each of these cytokines were observed in TLR4 mutant mice. Further, we utilized quantitative reverse transcriptase-polymerase chain reaction (RT-PCR) to measure a number of intra-graft inflammatory mediators and found that there was also significantly less upregulation of TNFα, IL-6, IL-1β, and ICAM-1 mRNA in TLR4 mutant mice compared to controls. These data demonstrated that TLR4 mediates both systemic and intragraft inflammation in the after cold I/R in the setting of an experimental model of cardiac transplantation. We also observed significantly lower levels of serum troponin I in TLR4 mutant mice compared to wild-type controls 

TLR4 is expressed on multiple different donor and recipient cell types including leukocytes, endothelial cells, dendritic cells, and cardiomyocytes. In order to determine whether TLR4 signaling on donor or recipient cells contributes to the early inflammatory response in the setting of cold I/R, we performed transplants using TLR4 mutant mice as donors with TLR4 wild-type mice as recipients. In addition, we also performed transplants using TLR4 wild-type mice as donors with TLR4 mutant mice as recipients. Interestingly, if TLR4 signaling was absent in either donor or recipient cells, the observed inflammatory response was reduced. Our data implicate TLR4 as a key mediator of inflammation and myocardial injury after I/R and further suggest that TLR4 signaling on both graft and recipient cells contributes to systemic and intragraft inflammatory responses in the setting of cold I/R. Further study will be required to better understand the nature of the ligands that stimulate TLR4 in the setting of cold I/R injury. The intracellular pathways utilized by TLR4 to mediate inflammation and organ injury after cold I/R will also require further study.

## TLR4 AND ATHEROSCLEROSIS

Evidence is also accumulating for a potential role for TLRs, particularly TLR4, in the development of atherosclerosis. A possible role for TLR4 in atherogenesis was suggested when Xu and co-investigators made the observation that TLR4 is expression is abundant in both murine and human atherosclerotic plaques and that TLR4 appears to be upregulated in response to oxidized low-density lipoprotein [43]. It was subsequently demonstrated that genetic deficiency of either TLR4 or the adaptor MyD88 results in reduced development of plaque in atherosclerotic prone *Apoe*-/- mice despite persistent hypercholersterolemia [44]. Others have also observed that MyD88 plays a key role in murine models of atherosclerosis [45]. In another study, overexpression of TLR2 and TLR4 in the carotid arterial vessel wall in a rabbit model led to accelerated atherosclerosis in animals fed a high fat diet [46].

The results of these small animal studies have been supported by population studies examining the development of atherosclerosis in humans with polymorphisms (Asp299Gly and Thr399Ile) in TLR4, which impair its ability to elicit an inflammatory response[47]. Kiechl and co-investigators found that 55 out of 810 subjects carried the Asp299Gly TLR4 allele. Compared to those with wild-type TLR4, those carrying the Asp299Gly allele had lower levels of circulating inflammatory mediators and were more predisposed to severe bacterial infections, but had a lower risk of carotid atherosclerosis [48]. In a smaller case control study, Ameziane and colleagues found that the Asp299Gly allele was associated with decreased risk of acute coronary events, as well as a decrease in plasma fibrinogen and soluble ICAM-1 [49]. The Asp299Gly variant was also found to be associated with a lower risk of myocardial infarction [50] and other cardiovascular events [51] in patients receiving statins. Despite these findings, the results of multiple other studies have failed to demonstrate a consistent relationship between TLR4 polymorphisms and the development of atherosclerotic disease [52-59]. Based on these conflicting results, further careful evaluation of the role of TLR4 in atherosclerosis is warranted.

## TLR4 IN MALADAPTIVE VENTRICULAR REMODELING

Chronic ventricular remodeling after an ischemic insult can lead to the eventual development of congestive heart failure. Several well known factors, including β-adrenergic stimulation and activation of the renin-angiotensin-aldosterone system, contribute to the development of maladaptive remodeling after myocardial infarction. Inflammatory responses to necrotic tissue and matrix turnover are processes that also contribute to this process. Using a murine model involving permanent coronary ligation in wild-type and TLR4-mutant mice, Timmers and colleagues investigated whether TLR4 is involved in ventricular remodeling after myocardial infarction [60]. They demonstrated that mice deficient in TLR4 signaling exhibited reduced inflammation, as measured by macrophage infiltration and expression of pro-inflammatory cytokines, in the infarcted area. There was also significantly less matrix metalloproteinase-2 and -9 activity in the hearts of mutant mice. In the non-infarcted areas, there was less interstitial fibrosis and myocardial hypertrophy in the hearts of TLR4 mutant animals compared to those form wild-type animals. Furthermore, after infarction, hearts from TLR4-mutant animals demonstrated less change in ventricular end-diastolic volume and also exhibited preserved systolic function. 

The results of a number of other studies suggest that TLR4 plays an important role in maladaptive ventricular remodeling after ischemic injury or that TLR4 signaling is altered in the setting of heart failure. Increased TLR4 expression has been observed in murine hearts after ischemic injury and in human heart tissue from patients with idiopathic dilated cardiomyopathy [61]. Others have found that surface TLR4 expression levels are higher in monocytes from patients that have suffered a recent acute myocardial and that these cells produce greater amounts of cytokines compared to those from health human controls [62]. Further, monocytes from patients that went on to develop heart failure after suffering an MI had higher levels of TLR4 surface expression and produced greater amounts of cytokines, including IL-6, TNF, and granulocyte/macrophage-colony stimulating factor (GM-CSF), upon LPS stimulation compared to those from patients who did not develop heart failure after MI. 

Based on the observation that NF-κB activation can lead to cardiomyocyte hypertrophy and that TLR4 stimulation can result in NF-κB activation, Ha and co-investigators hypothesized that TLR4 signaling might play a role in cardiomyocyte hypertrophy in an *in vivo* model [63]. In order to test their hypothesis, the authors employed a model involving aortic banding to induce pressure overload. Interestingly, significantly less myocardial hypertrophy and less NF-κB activation was observed in TLR4 deficient mice compared to wild-type controls. Another recent study has also demonstrated that improved ventricular function and less oxidative stress, inflammation, and apoptosis in TLR4 deficient mice compared to wild-type controls in the setting of doxorubicin-induced cardiomyopathy in mice [64]. Collectively, these findings suggest that TLR4 not only plays a role in adverse ventricular remodeling after ischemic injury, but that TLR4 also mediates maladaptive responses in response to other stimuli as well.

## CONCLUSIONS

In summary, the etiology of myocardial I/R injury is multifactorial, but activation of the innate immune system and the resultant inflammatory response are important components of I/R injury. Toll-like receptor 4 (TLR4), which was initially identified as the sensor for bacterial lipopolysaccharide (LPS), has also been shown to serve as a sensor for endogenous molecules released from damaged or ischemic tissues. The studies reviewed here demonstrate that TLR4 not only plays a role as a mediator of cardiac dysfunction in sepsis, but also serves as a key mediator of myocardial injury and inflammation in the setting of I/R. In addition, TLR4 signaling contributes to the maladaptive ventricular remodeling that may occur after ischemic myocardial injury. Further study will be required to identify the ligands that stimulate TLR4 in the setting of myocardial I/R injury and to better understand intracellular pathways that mediate TLR4 signaling after I/R. A better understanding of these pathways may lead to the development of therapeutic strategies to mitigate myocardial I/R injury.

## Figures and Tables

**Table 1:** TLR4 as a Mediator of Myocardial Ischemia/Reperfusion Injury

Study	Year	Model	Technique	TLR4	Findings
Oyama *et al.* [37]	2004	Warm ischemia	Temporary LAD occlusion; 1 hr ischemia, 24 hrs reperfusion	Genetic deficiency of TLR4	Reduced infarct size, inflammatory infiltrate, lipid peroxidation, and complement deposition in the absence of TLR4 signaling
Chong *et al.* [38]	2004	Warm ischemia	Temporary LAD occlusion; 1 hr ischemia, up to 2 hrs reperfusion	Genetic deficiency of TLR4	Reduced infarct size, MAP kinase activation, pro-inflammatory cytokine mRNA production in mice deficient in TLR4 signaling
Hua *et al.* [41]	2005	Warm ischemia	Temporary LAD occlusion; 45 min ischemia, 4 hrs reperfusion	Inhibition of MyD88 using dominant negative genetic construct	Reduced infarct size, NF-κB activation, cardiomyocyte apoptosis, increased Akt phosphorylation and BCL-2 expression after inhibition of MyD88 signaling
Shimamoto *et al.* [40]	2006	Warm ischemia	Temporary LAD occlusion; 30 min ischemia, upto 2 hrs reperfusion	Blockade of TLR4 signaling with a soluble inhibitor	Reduced infarct size, MAP kinase activation, NF-κB activation, pro-inflammatory cytokine mRNA production after inhibition of TLR4 with a soluble inhibitor, Eritoran
Hua *et al.* [39]	2007	Warm ischemia	Temporary LAD occlusion; 45 min ischemia, 4 hrs reperfusion	Genetic deficiency of TLR4, Pharmacologic inhibition of PI3/Akt signaling	Pharmacologic inhibition of PI3K/Akt signaling abrogates the reduction of myocardial injury observed in the absence of TLR4 signaling
Kaczorowski *et al.* [42]	2007	Cold ischemia	Global ischemia in cold preservation solution followed by heterotopic transplantation; 2 hrs cold ischemia, 3 or 24 hrs reperfusion	Genetic deficiency of TLR4	Reduced systemic and intragraft inflammatory mediators, reduced NF-κB activation, and reduced myocardial injury as measured by serum troponins in the absence of TLR4 signaling
